# Comparison of circular and elliptical geometric models with optimal two-dimensional imaging view for Doppler quantification of mitral regurgitant volume in older patients

**DOI:** 10.3389/fcvm.2026.1843639

**Published:** 2026-08-31

**Authors:** Wu-Gang Wang, Ning Zhang, Miao-Miao Pei, Xue-Jia Guo, Gai-Qin Liu, Yan-Dong Deng

**Affiliations:** Department of Ultrasound, The First Hospital of Hebei Medical University, Shijiazhuang, Hebei, China

**Keywords:** Doppler volumetric method, effective regurgitant orifice area, mitral regurgitation, older patients, regurgitant volume, three-dimensional echocardiography

## Abstract

**Background:**

Doppler volumetric quantification of mitral regurgitation (MR) depends on accurate stroke-volume estimation, but simplified assumptions regarding mitral annular area may introduce view-dependent error, particularly in older patients. This study evaluated whether circular versus elliptical annular models and standard two-dimensional (2D) imaging views influence Doppler-derived regurgitant volume (RVol) and effective regurgitant orifice area (EROA), using three-dimensional (3D) echocardiographic reference measures.

**Methods:**

In this retrospective study, 286 older patients with MR in sinus rhythm underwent transthoracic echocardiography with 2D imaging and pulsed-wave and continuous-wave Doppler, transthoracic 3D color Doppler imaging, and three-dimensional transesophageal echocardiography (3D TEE) for mitral annular imaging. Six prespecified combinations of 2D view and geometric model were used to estimate mitral annular cross-sectional area (CSA), RVol, and EROA. These included three circular models based on the parasternal long-axis (PLAX), apical four-chamber (A4C), or apical two-chamber (A2C) view and three elliptical models combining pairs of these views. Reference measures included 3D planimetric EROA (EROA₃D) and 3D-derived RVol (RVol₃D), calculated as EROA₃D multiplied by the MR velocity–time integral (MR VTI). Agreement was assessed using Bland–Altman analysis.

**Results:**

Mitral annular diameters differed among views (A4C > A2C > PLAX; *P* < 0.001). Compared with 3D TEE planimetry, elliptical CSA estimates showed smaller biases and narrower limits of agreement than circular estimates, with the A4C + A2C elliptical model showing the most favorable agreement. For the primary outcomes, the PLAX circular strategy underestimated both RVol and EROA, with biases of −10.8 mL and −0.080 cm^2^, respectively (both *P* < 0.001). The A4C + A2C elliptical strategy showed near-zero bias and the narrowest limits of agreement for both RVol (bias, −0.6 mL; *P* = 0.317) and EROA (bias, −0.005 cm^2^; *P* = 0.209), with consistent performance across MR mechanisms and severity strata.

**Conclusions:**

In older patients, Doppler-based MR quantification is sensitive to mitral annular geometric modeling and 2D view selection. An elliptical model integrating A4C and A2C diameters showed the most consistent agreement with 3D reference measures and may improve the standardization of Doppler-based MR assessment.

## 1 Introduction

Mitral regurgitation (MR) remains one of the most prevalent valvular disorders in older adults and is associated with progressive chamber remodeling, heart failure symptoms, atrial arrhythmias, functional decline, and excess mortality when significant regurgitation is left untreated (1). Accurate grading of MR severity is therefore central to therapeutic decision-making and longitudinal surveillance in older patients, particularly as surgical repair and transcatheter edge-to-edge repair have expanded across a broader spectrum of primary and secondary MR phenotypes in aging populations (2, 3). Contemporary imaging practice has increasingly emphasized quantitative assessment and multiparametric integration, supported by advances in three-dimensional (3D) echocardiography and transesophageal echocardiography (TEE) workflows (4–6). Despite these developments, echocardiographic quantification of MR remains technically challenging because multiple steps in the measurement chain are sensitive to anatomical complexity and acquisition conditions, which may be especially relevant in older patients with more complex valvular and ventricular remodeling (7, 8). 3D approaches, including 3D vena contracta area (VCA) and planimetric effective regurgitant orifice area (EROA), have been increasingly investigated as methods that reduce reliance on geometric assumptions inherent to two-dimensional (2D) techniques and may improve discrimination of clinically important MR severity thresholds (9, 10). However, 2D pulsed-wave (PW) Doppler–based volumetric methods remain widely used in routine practice because they rely on universally available measurements and are conceptually aligned with conservation of mass. In this framework, regurgitant volume is derived from the difference between transmitral and LVOT stroke volumes, making the accuracy of stroke-volume calculations a principal determinant of MR quantification performance (11).

A critical but underappreciated source of error in Doppler volumetric MR assessment lies in the estimation of mitral annular cross-sectional area (CSA) (12). The mitral annulus is non-circular, non-planar, and dynamically deforming; consequently, single-diameter circular assumptions may introduce systematic bias, and view-dependent diameter selection can further amplify variability because CSA scales nonlinearly with diameter. This issue may be particularly relevant in older patients, in whom age-related valvular degeneration, annular remodeling, and heterogeneity in MR mechanism may further affect annular geometry (13). Increasing evidence from 3D TEE–based studies and biomechanical analyses underscores that annular geometry and dynamics are clinically relevant and may differ by MR mechanism, providing a rationale for reassessing how the annulus is modeled when quantitative Doppler methods are applied (14). At the same time, advances in 3D mitral valve analysis, ranging from comprehensive TEE screening workflows to emerging automated segmentation and modeling, support the feasibility of acquiring high-quality 3D datasets for method validation in contemporary practice (15).

Against this background, the present study focused on older patients with MR and integrated transthoracic 2D Doppler volumetric quantification with 3D color Doppler planimetry and 3D TEE annular planimetry to evaluate how circular versus elliptical annular modeling and standard 2D view selection influence Doppler-derived MR indices. By anchoring comparisons to 3D-derived reference measures while maintaining clinically implementable acquisition protocols, this work aims to inform practical standardization of annular modeling choices in Doppler-based MR quantification in older adults across contemporary echocardiography workflows.

## 2 Methods

### 2.1 Study design

This retrospective observational study consecutively enrolled older patients with clinically diagnosed MR who underwent comprehensive transthoracic echocardiography incorporating 2D PW Doppler assessment and 3D color Doppler imaging at our institution between January 2023 and December 2025. Eligible participants were required to (1) be aged 60 years or older and have echocardiographically confirmed MR; (2) be in stable sinus rhythm with adequate image quality permitting reliable acquisition of mitral annular diameters from standard 2D views, including the parasternal long-axis (PLAX), apical four-chamber (A4C), and apical two-chamber (A2C) views, as well as left ventricular outflow tract (LVOT) measurements; and (3) undergo 3D color Doppler–guided planimetric EROA assessment to provide the reference for Doppler-derived regurgitant volume quantification. Patients were excluded if they had atrial fibrillation or frequent premature beats that precluded cycle-averaged Doppler quantification, concomitant moderate-to-severe aortic regurgitation, any degree of mitral stenosis, congenital heart disease, prior mitral valve intervention, or suboptimal acoustic windows and/or limited breath-holding ability resulting in non-diagnostic 2D or 3D datasets. The study protocol was reviewed and approved by the ethics committee of our hospital. Informed consent was obtained from all subjects. All procedures were conducted in accordance with relevant guidelines and regulations and complied with the ethical principles of the Declaration of Helsinki. All data were handled confidentially, and personal identifiers were removed prior to analysis to protect participant privacy.

### 2.2 Echocardiography platform, image acquisition, and offline analysis

Comprehensive echocardiographic examinations were performed using a commercially available ultrasound system (Philips EPIQ CVx, Philips Healthcare). TTE was acquired with phased-array and matrix-array transducers (S5-1 and X5-1), and 3D TEE was obtained using a dedicated 3D TEE probe (X8-2t). All datasets were exported in Digital Imaging and Communications in Medicine (DICOM) format and analyzed offline using QLAB software (version 10.1; Philips Healthcare). 3D color Doppler quantification was conducted with the Cardiac 3D Quantification (3DQ) module. For 2D TTE acquisition, standard PLAX, A4C, and A2C views were obtained. In each view, the mitral annular diameter was measured at mid-diastole, corresponding to maximal mitral leaflet opening, from the inner edge of one annular hinge point to the inner edge of the opposing hinge point. The same cardiac-cycle phase was used across the three views within each patient to minimize temporal variation in annular dimensions. PW Doppler recordings were acquired to obtain the mitral inflow velocity–time integral (MV VTI) and the LVOT velocity–time integral (LVOT VTI). Doppler beam alignment was optimized with angle correction, maintaining an insonation angle of <20° relative to the direction of blood flow whenever feasible. Continuous-wave (CW) Doppler was used to record the MR jet, from which peak MR velocity and the MR velocity–time integral (MR-VTI) were measured.

For TTE 3D color Doppler acquisition, full-volume datasets encompassing the MR jet were obtained by stitching four consecutive cardiac cycles. Color Doppler settings were standardized with a Nyquist limit of 40–60 cm/s, and acquisition frame rates were maintained at ≥10 frames per second to ensure temporal adequacy for subsequent multiplanar reconstruction. Offline 3D analysis was performed in QLAB using the 3DQ workflow: multiplanar reconstruction was used to visualize the regurgitant jet in orthogonal planes and to facilitate quantitative measurements as prespecified. 3D TEE imaging for mitral annular assessment was performed by acquiring a dedicated full-volume dataset in a mid-esophageal four-chamber orientation to ensure complete visualization of the mitral annulus. During offline analysis, the 3D dataset was interactively cropped and rotated to obtain a plane that clearly delineated the annular contour. The mitral annular CSA was then measured directly using planimetry on the optimally aligned annular plane, serving as the 3D annular reference measurement for geometric model comparisons. All 2D and 3D measurements were performed offline by the same observer according to the prespecified measurement protocol.

### 2.3 Variables and computational methods

**Figure 1:**
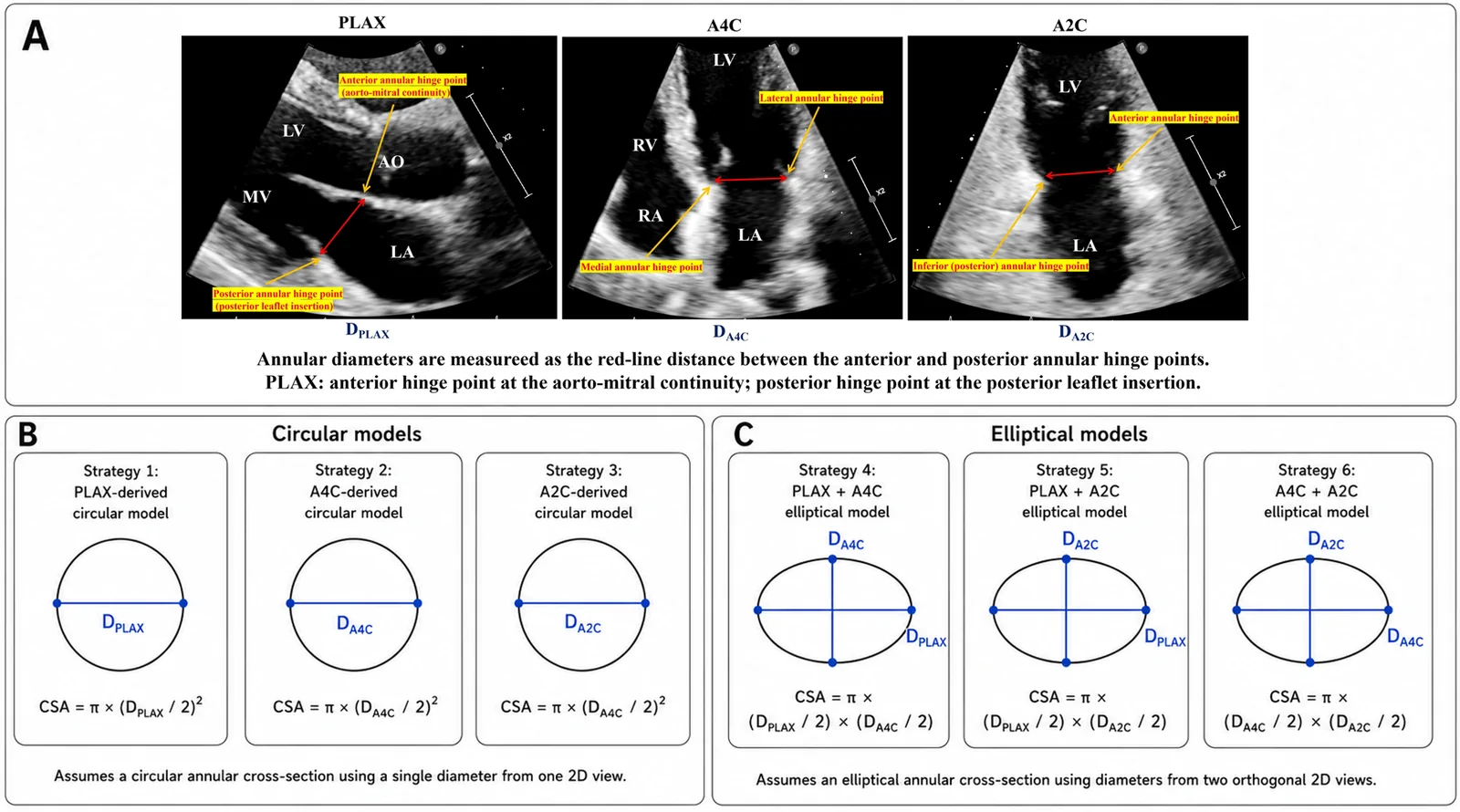
Schematic illustration of the six two-dimensional strategies used to estimate mitral annular cross-sectional area. **(A)** Mitral annular diameters were measured in the parasternal long-axis (PLAX), apical four-chamber (A4C), and apical two-chamber (A2C) views at mid-diastole. Each diameter was defined as the straight-line distance between the opposing annular hinge points. In the PLAX view, the measurement extended from the anterior hinge point at the aorto-mitral continuity to the posterior hinge point at the posterior leaflet insertion. **(B)** Circular models estimated cross-sectional area from a single-view diameter as pi multiplied by the square of one-half of the diameter. **(C)** Elliptical models estimated cross-sectional area from paired diameters obtained from two different views as pi multiplied by one-half of the first diameter and one-half of the second diameter. A4C, apical four-chamber; A2C, apical two-chamber; CSA, cross-sectional area; PLAX, parasternal long-axis.

Mitral annular and LVOT CSAs were defined using prespecified geometric models. All mitral annular diameters used to construct the six prespecified geometric models were obtained at the standardized mid-diastolic frame corresponding to maximal mitral leaflet opening, as described above. For the mitral annulus, the circular model was calculated as: CSA_MV = π  ×  a^2^/4, where a denotes the mitral annular diameter measured from a single 2D view. Three circular-model variants were generated by assigning a from the PLAX, A4C, or A2C view, respectively. The elliptical model was calculated as: CSA_MV = π × a × b/4, where a and b represent paired mitral annular diameters obtained from two 2D views. Three elliptical-model variants were constructed using PLAX + A4C, PLAX + A2C, and A4C + A2C diameter combinations. A schematic illustration of the six prespecified 2D strategies is provided in Figure 1. The LVOT area was modeled as circular and computed as: CSA_LVOT = π × d^2^/4, where d is the LVOT diameter. Mitral regurgitant volume and EROA were quantified using the PW Doppler volumetric (flow-difference) method based on the continuity principle. Stroke volume (SV) was calculated as: SV = CSA   ×   VTI. Specifically, forward mitral stroke volume was computed as: SV_MV = CSA_MV × MV VTI, and LVOT stroke volume was computed as: SV_LVOT = CSA_LVOT   ×   LVOT VTI. Doppler-derived regurgitant volume (RVol) was defined as the flow difference between mitral and LVOT stroke volumes: RVol_Doppler = SV_MV−SV_LVOT. Doppler-derived EROA was then calculated as: EROA_Doppler = RVol_Doppler/MR-VTI, where MR-VTI was obtained from CW Doppler interrogation of the regurgitant jet. For all Doppler-derived parameters, measurements were averaged over three cardiac cycles to reduce beat-to-beat variability.

3D color Doppler planimetry served as the reference approach for EROA. Using QLAB (version 10.1) in the 3DQ workflow, multiplanar reconstruction was performed to align the measurement plane perpendicular to the MR jet. The plane was advanced along the jet to identify the narrowest jet cross-section, and the minimal regurgitant jet area was manually traced to derive EROA_3D. A 3D reference regurgitant volume was subsequently computed as: RVol_3D = EROA_3D × MR-VTI, using the MR-VTI obtained from the same echocardiographic examination. The mitral annular CSA was measured by direct planimetry on the optimally aligned en face annular plane at mid-diastole, corresponding to maximal mitral leaflet opening. This measurement was therefore phase-matched to the 2D annular measurements and was denoted CSA_MV (3D TEE). This direct 3D annular measurement was used as the reference to assess the bias and agreement of the 2D-derived circular and elliptical CSA estimates across imaging-view combinations.

### 2.4 Outcomes

The primary measurement outcomes were the agreement of Doppler-derived mitral regurgitant volume (RVol_Doppler) and EROA (EROA_Doppler) with their respective 3D reference measures. The six prespecified “2D view   ×   geometric model” strategies, comprising three circular and three elliptical approaches, were evaluated in parallel. No individual strategy was designated *a priori* as the primary strategy, and no formal hierarchical testing sequence was applied.

For regurgitant volume, RVol_Doppler was compared with the 3D color Doppler reference, RVol_3D, and agreement was summarized using mean bias (RVol_Doppler−RVol_3D) and Bland–Altman 95% limits of agreement (LoA). For EROA, EROA_Doppler was compared with the 3D planimetric reference, EROA_3D, using mean bias (EROA_Doppler−EROA_3D) and Bland–Altman 95% LoA. The criteria for identifying the best-performing strategy were prespecified as the smallest absolute bias together with the narrowest LoA. Thus, the evaluation criteria, rather than a specific geometric strategy, were defined before the comparative analyses.

Secondary outcomes examined geometric and computational contributors to measurement differences. Mitral annular CSA estimates derived from each 2D strategy (CSA_MV) were compared with the direct 3D TEE planimetric annular area, CSA_MV (3D TEE), using mean bias and Bland–Altman 95% LoA. Inter-view differences in mitral annular diameters among the PLAX, A4C, and A2C views were also assessed. Intermediate Doppler components, including SV_MV, SV_LVOT, and SV_MV−SV_LVOT, were summarized to facilitate interpretation of discrepancies between Doppler-derived and 3D reference regurgitant volumes.

Subgroup analyses were prespecified according to MR mechanism. The primary agreement analyses for RVol and EROA were repeated separately in patients with functional MR (FMR) and primary/organic MR (PMR) to assess whether the effects of geometric assumptions and imaging-view selection differed by mechanism. Additional stratified analyses according to MR severity, defined using the 3D reference measures, were conducted to evaluate whether bias and agreement varied across severity strata.

### 2.5 Statistical analysis

All statistical analyses were performed using IBM SPSS Statistics, version 28.0 (IBM Corp., Armonk, NY, USA) and R software, version 4.3.2 (R Foundation for Statistical Computing, Vienna, Austria). Continuous variables are presented as mean ± standard deviation (SD), and categorical variables as number (percentage). Between-group comparisons (FMR vs. PMR) for continuous variables were conducted using the independent-samples t test, and categorical variables were compared using the *χ*^2^ test or Fisher's exact test, as appropriate. Inter-view differences in mitral annular diameters (PLAX, A4C, and A2C) were assessed using repeated-measures analysis of variance (ANOVA), with Bonferroni-adjusted paired comparisons. Agreement between Doppler-derived and 3D reference measurements (RVol and EROA), and between 2D model–derived CSA estimates and 3D TEE annular CSA, was evaluated using Bland–Altman analysis, reporting mean bias (Doppler/2D model minus reference) and 95% limits of agreement. The six prespecified geometric strategies were evaluated in parallel as exploratory comparisons. For each strategy, mean bias was tested against zero using a one-sample t test of the paired differences. Pearson correlation coefficients were calculated to assess linear associations between Doppler-derived estimates and 3D reference measures and between intermediate Doppler components (SV_MV−SV_LVOT) and RVol_3D. All tests were two-sided, and a *P* value <0.05 was considered statistically significant.

## 3 Results

### 3.1 Study population and enrollment

Between January 2023 and December 2025, a total of 317 consecutive older patients with echocardiographically confirmed MR who underwent a comprehensive echocardiographic protocol, including TTE (2D imaging with PW/CW Doppler), TTE 3D color Doppler acquisition, and 3D TEE annular imaging, were screened for eligibility. After applying the prespecified criteria, 31 patients were excluded, and 286 patients were ultimately included in the final analysis. The reasons for exclusion were as follows: atrial fibrillation (*n* = 12), frequent premature beats precluding reliable cycle-averaged Doppler quantification (*n* = 5), concomitant mitral stenosis (*n* = 4), concomitant moderate-to-severe aortic regurgitation (*n* = 4), congenital heart disease (*n* = 2), prior mitral valve intervention/surgery (*n* = 2), and non-diagnostic echocardiographic datasets due to suboptimal acoustic windows and/or inadequate breath-holding (*n* = 2). The final study cohort therefore comprised 286 older patients, who were subsequently classified by MR mechanism into FMR (*n* = 142) and PMR (*n* = 144) for prespecified subgroup analyses.

### 3.2 Baseline clinical and echocardiographic characteristics

Among the 286 older patients included in the final analysis (FMR, *n* = 142; PMR, *n* = 144), the FMR group was older than the PMR group (69.9 ± 8.8 vs. 65.4 ± 9.4 years; t = 4.18; *P* < 0.001). Sex distribution was comparable between groups (male sex: 61.3% vs. 57.6%; *χ*^2^ = 0.39; *P* = 0.532). Patients with FMR had a higher BMI (26.2 ± 3.4 vs. 24.3 ± 3.4 kg/m^2^; t = 4.90; *P* < 0.001), whereas BSA did not differ significantly (1.83 ± 0.14 vs. 1.81 ± 0.15 m^2^; t = 1.27; *P* = 0.207). Echocardiographically, the FMR group demonstrated greater left ventricular remodeling, with larger LVEDD and LVESD (LVEDD: 59.8 ± 7.1 vs. 55.2 ± 5.8 mm, t = 5.95, *P* < 0.001; LVESD: 46.4 ± 7.5 vs. 36.6 ± 6.4 mm, t = 11.87, *P* < 0.001), and lower systolic function (LVEF: 43.7 ± 7.6% vs. 59.2 ± 6.9%; t = −18.10; *P* < 0.001). LA diameter was slightly larger in the FMR group (43.7 ± 6.1 vs. 42.0 ± 6.3 mm; t = 2.26; *P* = 0.024). Consistently, PASP was higher in FMR than in PMR (43.1 ± 11.9 vs. 36.2 ± 9.7 mmHg; t = 5.37; *P* < 0.001) (Table 1).

**Table 1:** Baseline characteristics of the study cohort stratified by MR mechanism.

Variable	FMR (*n* = 142)	PMR (*n* = 144)	Test statistic	*P* value
Age, years	69.9 ± 8.8	65.4 ± 9.4	t = 4.18	<0.001
Male sex, *n* (%)	87 (61.3)	83 (57.6)	*χ*^2^ = 0.39	0.532
BMI, kg/m^2^	26.2 ± 3.4	24.3 ± 3.4	t = 4.90	<0.001
BSA, m^2^	1.83 ± 0.14	1.81 ± 0.15	t = 1.27	0.207
LVEDD, mm	59.8 ± 7.1	55.2 ± 5.8	t = 5.95	<0.001
LVESD, mm	46.4 ± 7.5	36.6 ± 6.4	t = 11.87	<0.001
LVEF, %	43.7 ± 7.6	59.2 ± 6.9	t = −18.10	<0.001
LA diameter, mm	43.7 ± 6.1	42.0 ± 6.3	t = 2.26	0.024
PASP, mmHg	43.1 ± 11.9	36.2 ± 9.7	t = 5.37	<0.001

BMI, body mass index; BSA, body surface area; FMR, functional mitral regurgitation; PMR, primary/organic mitral regurgitation; LA, left atrium/left atrial; LVEDD, left ventricular end-diastolic diameter; LVESD, left ventricular end-systolic diameter; LVEF, left ventricular ejection fraction; MR, mitral regurgitation; PASP, pulmonary artery systolic pressure.

### 3.3 Data availability and measurement feasibility

Overall feasibility of multimodality quantification was high (Table 2). Mitral annular diameters were obtainable in 96.2%–99.0% of patients across the three standard 2D views, and Doppler VTIs were available in nearly all participants (MV VTI: 100.0%; LVOT VTI: 99.3%; MR-VTI: 98.6%). Prespecified TTE 3D acquisition criteria were met in most datasets, with 95.5% achieving a frame rate ≥10 fps and 97.2% acquired with a Nyquist limit of 40–60 cm/s. Direct planimetric measurement of mitral annular CSA on 3D TEE was feasible in 94.1% of patients. None of the availability or acquisition-quality metrics differed significantly between the FMR and PMR groups (all *P* ≥ 0.486), supporting comparable measurement feasibility across MR mechanisms.

**Table 2:** Availability of key measurements, acquisition quality metrics, and distribution of reference indices.

Variable	Overall (*n* = 286)	FMR (*n* = 142)	PMR (*n* = 144)	Test statistic	*P* value
Data availability/feasibility, *n* (%)					
PLAX mitral annular diameter available	280 (97.9)	139 (97.9)	141 (97.9)	χ^2^ = 0.00	0.986
A4C mitral annular diameter available	283 (99.0)	141 (99.3)	142 (98.6)	χ^2^ = 0.32	0.570
A2C mitral annular diameter available	275 (96.2)	136 (95.8)	139 (96.5)	χ^2^ = 0.11	0.741
MV VTI available	286 (100.0)	142 (100.0)	144 (100.0)	—	—
LVOT VTI available	284 (99.3)	141 (99.3)	143 (99.3)	χ^2^ = 0.00	0.992
MR-VTI available	282 (98.6)	140 (98.6)	142 (98.6)	χ^2^ = 0.00	0.989
3D TEE frame rate ≥10 fps (met)	273 (95.5)	136 (95.8)	137 (95.1)	χ^2^ = 0.07	0.796
3D TEE Nyquist 40–60 cm/s (met)	278 (97.2)	139 (97.9)	139 (96.5)	χ^2^ = 0.49	0.486
3D TEE annular CSA measurable	269 (94.1)	134 (94.4)	135 (93.8)	χ^2^ = 0.05	0.826
Quantitative indices, mean ± SD					
MR-VTI, cm	145.5 ± 31.3	141.0 ± 32.0	150.0 ± 30.0	t = −2.45	0.015
MV VTI, cm	19.3 ± 4.4	18.5 ± 4.2	20.1 ± 4.5	t = −3.11	0.002
LVOT VTI, cm	18.8 ± 3.7	19.0 ± 3.8	18.7 ± 3.7	t = 0.68	0.499
EROA_3D, cm^2^	0.345 ± 0.126	0.330 ± 0.120	0.360 ± 0.130	t = −2.03	0.043
RVol_3D, mL	47.5 ± 19.1	46.0 ± 18.0	49.0 ± 20.0	t = −1.33	0.183

A2C, apical two-chamber; A4C, apical four-chamber; CSA, cross-sectional area; EROA, effective regurgitant orifice area; EROA_3D, three-dimensional color Doppler planimetric effective regurgitant orifice area; FMR, functional mitral regurgitation; fps, frames per second; LVOT, left ventricular outflow tract; MR, mitral regurgitation; MR-VTI, mitral regurgitation velocity–time integral; MV, mitral valve; MV VTI, mitral inflow velocity–time integral; PLAX, parasternal long-axis; PMR, primary/organic mitral regurgitation; RVol_3D, three-dimensional reference regurgitant volume calculated as EROA_3D × MR-VTI; TEE, transesophageal echocardiography; TTE, transthoracic echocardiography; VTI, velocity–time integral.

Reference and supporting quantitative indices are also summarized in Table 2. In the overall cohort, mean EROA_3D was 0.345 ± 0.126 cm^2^ and mean RVol_3D was 47.5 ± 19.1 mL. Compared with PMR, FMR was associated with lower MR-VTI (141.0 ± 32.0 vs. 150.0 ± 30.0 cm; *P* = 0.015) and lower MV VTI (18.5 ± 4.2 vs. 20.1 ± 4.5 cm; *P* = 0.002), whereas LVOT VTI was similar between groups (*P* = 0.499). EROA_3D was modestly smaller in FMR than in PMR (*P* = 0.043), while RVol_3D did not differ significantly by mechanism (*P* = 0.183).

### 3.4 Inter-view differences in 2D mitral annular diameters

**Figure 2:**
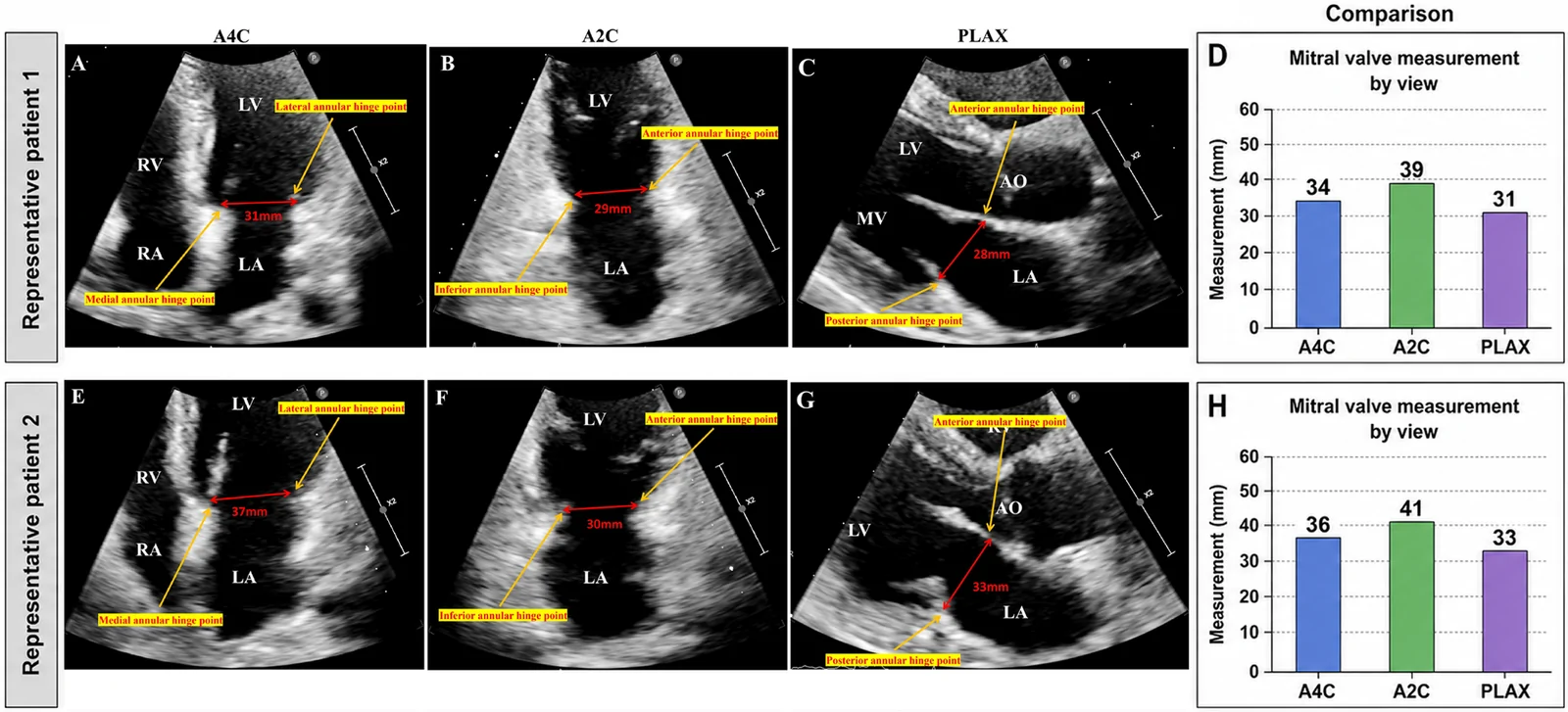
Representative inter-view variability in mitral annular diameter measurements. Mitral annular diameters were measured in the apical four-chamber (A4C), apical two-chamber (A2C), and parasternal long-axis (PLAX) views in two representative patients. Panels **A–C** and **E–G** show the corresponding measurements, whereas panels **D** and **H** summarize the measurements graphically. Blue calipers indicate the straight-line distance between opposing mitral annular hinge points.

In the paired analysis including 270 patients with complete annular diameter measurements across all three standard 2D views, mitral annular diameters differed significantly by imaging plane [repeated-measures ANOVA: F(2,538) = 92.4; *P* < 0.001] (Table 3). The annular diameter measured in the A4C view was the largest (36.2 ± 4.7 mm), followed by A2C (35.4 ± 4.6 mm), whereas PLAX yielded the smallest diameter (34.8 ± 4.5 mm). Representative patient-level examples of these inter-view differences are shown in Figure 2. Pairwise comparisons confirmed significant inter-view differences after Bonferroni adjustment, with A4C exceeding PLAX by 1.40 ± 2.45 mm (t = 9.38; *P* < 0.001), A2C exceeding PLAX by 0.60 ± 2.20 mm (t = 4.48; *P* < 0.001), and A4C exceeding A2C by 0.80 ± 2.35 mm (t = 5.59; *P* < 0.001).

**Table 3:** Comparison of mitral annular diameters across 2D imaging views.

Variable	Mean ± SD (mm)	Test statistic	*P* value
PLAX annular diameter	34.8 ± 4.5		
A4C annular diameter	36.2 ± 4.7		
A2C annular diameter	35.4 ± 4.6		
Overall difference across views (repeated-measures ANOVA)	—	F(2, 538) = 92.4	<0.001
Pairwise comparisons (paired t-tests, Bonferroni-adjusted)			
A4C−PLAX	1.40 ± 2.45	t = 9.38	<0.001
A2C−PLAX	0.60 ± 2.20	t = 4.48	<0.001
A4C−A2C	0.80 ± 2.35	t = 5.59	<0.001

A2C, apical two-chamber; A4C, apical four-chamber; PLAX, parasternal long-axis.

### 3.5 CSA accuracy: agreement of 2D geometric models with the 3D TEE annular reference

In 269 older patients with measurable 3D TEE annular planimetry, agreement between 2D-derived mitral annular CSA and the 3D TEE reference varied across geometric strategies (Table 4). Among the circular models, PLAX showed the largest observed underestimation (bias −0.67 cm^2^; 95% LoA −1.53 to 0.19; *P* < 0.001), whereas A4C showed the smallest absolute bias (−0.05 cm^2^; 95% LoA −0.76 to 0.66; *P* = 0.023). The elliptical strategies generally showed smaller absolute biases and narrower LoA than the single-diameter circular strategies. The A4C + A2C elliptical model showed the most favorable observed agreement profile with 3D TEE, with a bias of −0.10 cm^2^ and the narrowest LoA of −0.67 to 0.47 cm^2^. Overall, the observed agreement patterns varied according to both the geometric assumption and the imaging views used for annular diameter measurement, with the two-apical-view elliptical model showing closer agreement with the 3D annular reference.

**Table 4:** Agreement between 2D-derived mitral annular CSA estimates and 3D TEE planimetric CSA.

2D strategy (view × model)	Bias, cm^2^ (mean ± SD)	95% LoA, cm^2^ (lower to upper)	Test statistic, one-sample t test of paired differences	*P* value	Agreement summary
Circular model					
PLAX (CSA = πa^2^/4)	−0.67 ± 0.44	−1.53 to 0.19	t = −24.91	<0.001	Systematic underestimation; wide LoA
A4C (CSA = πa^2^/4)	−0.05 ± 0.36	−0.76 to 0.66	t = −2.28	0.023	Minimal bias; moderate LoA
A2C (CSA = πa^2^/4)	−0.34 ± 0.40	−1.12 to 0.44	t = −13.91	<0.001	Underestimation; moderate–wide LoA
Elliptical model					
PLAX + A4C (CSA = πab/4)	−0.18 ± 0.31	−0.79 to 0.43	t = −9.50	<0.001	Mild underestimation; relatively narrow LoA
PLAX + A2C (CSA = πab/4)	−0.29 ± 0.33	−0.94 to 0.36	t = −14.39	<0.001	Underestimation; moderate LoA
A4C + A2C (CSA = πab/4)	−0.10 ± 0.29	−0.67 to 0.47	t = −5.65	<0.001	Small bias; narrowest LoA

A2C, apical two-chamber; A4C, apical four-chamber; CSA, cross-sectional area; LoA, limits of agreement; MV, mitral valve; PLAX, parasternal long-axis; TEE, transesophageal echocardiography.

### 3.6 Primary outcomes: accuracy of RVol and EROA across the six prespecified “2D view   ×   geometric model” combinations

Across the six prespecified strategies, Doppler-derived regurgitant volume (RVol_Doppler) showed varying degrees of bias relative to the 3D reference measure (RVol_3D) (Table 5). The PLAX-based circular model showed the largest observed underestimation, with a mean bias of −10.8 mL and 95% limits of agreement (LoA) from −38.2 to 16.6 mL (*P* < 0.001). The elliptical strategies generally showed smaller absolute biases and narrower LoA than the single-view circular strategies. According to the prespecified descriptive criteria, the A4C + A2C elliptical model had the most favorable observed agreement profile for RVol, with the smallest absolute bias (−0.6 mL; *P* = 0.317), the narrowest LoA (−19.8 to 18.6 mL), and the highest correlation with RVol_3D (r = 0.92). A similar pattern was observed for EROA. The PLAX-based circular model showed the largest observed underestimation relative to EROA_3D, with a mean bias of −0.080 cm^2%^ and 95% LoA from −0.256 to 0.096 cm^2^ (*P* < 0.001). The A4C + A2C elliptical model had the smallest absolute EROA bias (−0.005 cm^2^; *P* = 0.209), the narrowest LoA (−0.132 to 0.122 cm^2^), and a strong correlation with EROA_3D (r = 0.91).

**Table 5:** Agreement between Doppler-derived and 3D reference MR quantification across six strategies.

2D strategy (view × model)	RVol bias, mL (mean ± SD)	RVol 95% LoA, mL (lower to upper)	t value	*P* value	Pearson r (RVol_Doppler vs. RVol_3D)	EROA bias, cm^2^ (mean ± SD)	EROA 95% LoA, cm^2^ (lower to upper)	t value	*P* value	Pearson r (EROA_Doppler vs. EROA_3D)
Circular model										
PLAX (CSA_MV = πa^2^/4)	−10.8 ± 14.0	−38.2 to 16.6	−12.62	<0.001	0.78	−0.080 ± 0.090	−0.256 to 0.096	−14.55	<0.001	0.79
A4C (CSA_MV = πa^2^/4)	−1.2 ± 11.8	−24.3 to 21.9	−1.66	0.098	0.88	−0.010 ± 0.075	−0.157 to 0.137	−2.18	0.030	0.87
A2C (CSA_MV = πa^2^/4)	−5.6 ± 12.6	−30.3 to 19.1	−7.27	<0.001	0.83	−0.040 ± 0.080	−0.197 to 0.117	−8.18	<0.001	0.83
Elliptical model										
PLAX + A4C (CSA_MV = πab/4)	−2.8 ± 10.5	−23.4 to 17.8	−4.37	<0.001	0.90	−0.018 ± 0.070	−0.155 to 0.119	−4.21	<0.001	0.90
PLAX + A2C (CSA_MV = πab/4)	−4.1 ± 10.9	−25.5 to 17.3	−6.16	<0.001	0.88	−0.028 ± 0.072	−0.169 to 0.113	−6.36	<0.001	0.88
A4C + A2C (CSA_MV = πab/4)	−0.6 ± 9.8	−19.8 to 18.6	−1.00	0.317	0.92	−0.005 ± 0.065	−0.132 to 0.122	−1.26	0.209	0.91

*P* values were derived from tests of whether the mean bias differed from zero. Because the six strategies were evaluated in parallel as exploratory comparisons and no formal pairwise superiority testing was performed, these *P* values were not adjusted for multiplicity and should be interpreted descriptively. Relative strategy performance was assessed primarily according to the magnitude of bias and the width of the 95% LoA.

A2C, apical two-chamber; A4C, apical four-chamber; CSA, cross-sectional area; EROA, effective regurgitant orifice area; EROA_3D, three-dimensional color Doppler planimetric EROA; LoA, limits of agreement; MR, mitral regurgitation; MR-VTI, MR velocity–time integral; MV, mitral valve; MV VTI, mitral inflow velocity–time integral; PLAX, parasternal long-axis; RVol, regurgitant volume; RVol_3D, 3D reference regurgitant volume computed as EROA_3D × MR-VTI; SD, standard deviation; VTI, velocity–time integral.

### 3.7 Intermediate Doppler components

SV_MV differed across the six strategies [F(5,1335) = 58.6; *P* < 0.001], in parallel with the view- and model-dependent variation in estimated mitral annular CSA (Table 6). In contrast, SV_LVOT remained similar across strategies [F(5,1335) = 0.12; *P* = 0.988], consistent with use of the same LVOT geometric assumption and comparable LVOT VTI measurements. Accordingly, the between-strategy differences in RVol_Doppler paralleled variation in SV_MV and the flow-difference term, SV_MV−SV_LVOT. The flow-difference term was positively correlated with RVol_3D across all strategies (all *P* < 0.001), with the highest observed correlation for the A4C + A2C elliptical strategy (r = 0.92). Overall, the observed variation in Doppler-derived MR quantification was more closely associated with differences in mitral annular CSA and SV_MV estimates than with LVOT-derived forward-flow estimates.

**Table 6:** Intermediate Doppler components across six prespecified strategies and their relationship with the 3D reference regurgitant volume.

2D strategy (view × model)	SV_MV, mL (mean ± SD)	SV_LVOT, mL (mean ± SD)	SV_MV−SV_LVOT, mL (mean ± SD)	Pearson r vs. RVol_3D	*P* value (r)
Circular model					
PLAX (CSA_MV = πa^2^/4)	115.7 ± 24.3	79.0 ± 16.1	36.7 ± 18.0	0.78	<0.001
A4C (CSA_MV = πa^2^/4)	125.3 ± 25.0	79.0 ± 16.1	46.3 ± 16.5	0.88	<0.001
A2C (CSA_MV = πa^2^/4)	120.9 ± 24.7	79.0 ± 16.1	41.9 ± 17.2	0.83	<0.001
Elliptical model					
PLAX + A4C (CSA_MV = πab/4)	123.7 ± 24.6	79.0 ± 16.1	44.7 ± 15.8	0.90	<0.001
PLAX + A2C (CSA_MV = πab/4)	122.4 ± 24.5	79.0 ± 16.1	43.4 ± 16.2	0.88	<0.001
A4C + A2C (CSA_MV = πab/4)	125.9 ± 24.8	79.0 ± 16.1	46.9 ± 15.0	0.92	<0.001
Overall difference across strategies (repeated-measures ANOVA)	—	—	—	—	—
SV_MV across six strategies	—	—	F(5, 1335) = 58.6	—	<0.001
SV_LVOT across six strategies	—	—	F(5, 1335) = 0.12	—	0.988

A2C, apical two-chamber; A4C, apical four-chamber; CSA, cross-sectional area; LVOT, left ventricular outflow tract; MR, mitral regurgitation; PLAX, parasternal long-axis; RVol_3D, 3D reference regurgitant volume; SV, stroke volume; SV_LVOT, LVOT stroke volume (CSA_LVOT   ×   LVOT VTI); SV_MV, forward mitral stroke volume (CSA_MV × MV VTI); VTI, velocity–time integral.

### 3.8 Subgroup analyses by MR mechanism and severity

In the mechanism-stratified analyses, the relative agreement patterns across the six strategies were broadly similar in FMR and PMR (Supplementary Tables S1 and S2, 8). The PLAX-based circular model showed the largest observed underestimation and the widest LoA for both RVol and EROA in each subgroup. The elliptical strategies generally showed smaller absolute biases and narrower LoA, with the A4C + A2C elliptical strategy showing the most favorable observed agreement profile in both FMR and PMR. The LoA were modestly wider in FMR than in PMR for most strategies, indicating greater observed variability in the FMR subgroup. In the severity-stratified analysis based on EROA_3D, the A4C + A2C elliptical strategy maintained mean biases close to zero across the mild, moderate, and severe strata (Supplementary Table S3). The LoA were wider in the severe stratum, particularly for RVol, indicating greater absolute variability at higher MR severity. These exploratory analyses suggest that the overall agreement pattern was broadly consistent across MR mechanisms and severity strata.

## 4 Discussion

This study evaluated how mitral annular geometric assumptions and 2D imaging-view selection were associated with Doppler volumetric quantification of MR in older patients. Three principal findings emerged. First, multimodality measurements were feasible in most patients, with high availability of 2D annular diameters, Doppler VTIs, standardized TTE 3D color Doppler datasets, and 3D TEE annular planimetry. Second, mitral annular diameters differed across standard 2D views, with corresponding variation in CSA estimates, particularly when single-diameter circular assumptions were applied. Third, among the six prespecified strategies, the A4C + A2C elliptical model showed the most favorable observed agreement profile with both the 3D TEE annular CSA reference and the 3D reference regurgitant indices, with the smallest absolute bias and narrowest LoA for RVol and EROA. Collectively, in the present study, the observed between-strategy differences appeared to be primarily associated with annular geometry modeling and view-dependent diameter acquisition, whereas LVOT-derived forward-flow estimates remained comparatively stable across strategies.

A key observation was the ordered difference in mitral annular diameter across the standard 2D views (A4C > A2C > PLAX). This pattern is consistent with the non-circular, non-planar, and dynamically changing geometry of the mitral annulus (5, 16). In older patients, age-related valvular degeneration, annular remodeling, ventricular structural changes, and heterogeneity in MR mechanism may further increase geometric complexity (17). Because CSA under a circular assumption is proportional to the square of a single measured diameter, relatively small inter-view differences can translate into larger variation in estimated annular area and SV_MV. Consistent with this geometric relationship, the PLAX circular strategy showed the greatest negative bias, whereas the A4C circular strategy showed the smallest bias among the circular models. The elliptical strategies, particularly the A4C + A2C combination, showed narrower LoA and smaller absolute biases, suggesting that paired apical diameters may better represent annular asymmetry and reduce dependence on a single imaging plane. These observations are consistent with previous studies showing that single-diameter circular models are sensitive to view selection, whereas multi-diameter elliptical models generally show closer agreement with 3D annular measurements (18, 19).

The agreement patterns observed for annular CSA were also reflected in the RVol and EROA estimates. The PLAX circular strategy showed the largest observed underestimation for both RVol and EROA, together with relatively wide LoA, whereas the A4C + A2C elliptical strategy showed the smallest absolute biases and the narrowest LoA. These findings indicate that the choice of imaging view and geometric assumption may have clinically relevant implications, particularly for patients whose measurements are close to guideline-defined MR severity thresholds (20, 21). For primary MR, commonly used quantitative ranges include RVol values of 30–44 and 45–59 mL and EROA values of 0.20–0.29 and 0.30–0.39 cm^2^ for intermediate severity categories, whereas RVol ≥60 mL and EROA ≥0.40 cm^2^ support severe MR (22–24). Accordingly, a mean bias of −10.8 mL for RVol and −0.080 cm^2^ for EROA could potentially shift an estimate toward a lower severity category when the reference value lies near a classification boundary. The wide LoA further indicate that discrepancies may be larger in individual patients than suggested by the mean bias alone. However, the present study did not perform a formal reclassification analysis, and the frequency of actual category changes cannot be determined. Current American Society of Echocardiography (ASE) and the European Association of Cardiovascular Imaging (EACVI) recommendations advocate an integrated, multiparametric approach to MR grading that incorporates quantitative measures such as RVol and EROA rather than relying on a single parameter (25, 26). Within this framework, the A4C + A2C elliptical strategy may represent a potential practical refinement of the Doppler volumetric method, although prospective validation is required before its incorporation into routine guideline-based algorithms.

The intermediate-component analyses in this cohort of older patients provide internal consistency for the observed primary outcome patterns. SV_LVOT remained stable across strategies, whereas SV_MV varied significantly, mirroring the view- and model-dependent behavior of CSA_MV. Because RVol_Doppler is computed as SV_MV−SV_LVOT, variability in SV_MV becomes the dominant driver of strategy-specific RVol differences when LVOT measurements are relatively stable. Furthermore, the flow-difference term correlated strongly with RVol_3D across strategies, with the highest correlation again observed for the A4C + A2C elliptical strategy. These results support a parsimonious explanation: the principal determinant of Doppler volumetric error in older patients in this setting is annular CSA estimation rather than LVOT forward flow estimation. This observation is clinically relevant because it indicates where measurement standardization efforts are most likely to yield improvement, specifically in annular diameter acquisition and geometric modeling rather than in LVOT measurement, which is typically less variable because of its more circular geometry (27, 28). In older adults, such refinement may be especially important for improving the consistency and reliability of routine MR quantification.

Mechanism-stratified analyses showed a consistent ranking of strategies in both FMR and PMR among older patients, with modestly wider LoA in FMR for most strategies. This pattern is plausible given that FMR in older adults is often accompanied by annular dilatation, ventricular remodeling, and more complex or dynamically changing regurgitant orifices, all of which may increase measurement variability. The A4C + A2C elliptical strategy remained the best-performing approach in both mechanisms, suggesting that the benefit of incorporating two apical diameters is not limited to a single etiologic subtype. Similarly, when stratified by MR severity, near-zero mean bias was maintained across strata for the best-performing strategy, whereas LoA widened in more severe MR, an expected phenomenon because absolute measurement variability often scales with the magnitude of regurgitation (29, 30). These findings support the robustness of the selected strategy across mechanisms and across a clinically relevant severity spectrum in older adults, which is pertinent to contemporary management pathways that emphasize comprehensive and quantitative MR assessment as part of decision-making for intervention.

Several limitations should be acknowledged. First, this retrospective, single-center study included older patients in sinus rhythm with adequate image quality; therefore, the findings may reflect local acquisition practices and may not generalize to patients with atrial fibrillation, suboptimal acoustic windows, or other clinical populations. Second, RVol_3D was calculated as EROA_3D × MR-VTI and thus depended on both 3D planimetric EROA and CW Doppler MR-VTI. In addition, 3D color Doppler measurements remain sensitive to acquisition settings, temporal resolution, and jet characteristics. Third, 3D TEE annular CSA was not measurable in all patients, and missing data related to acoustic shadowing, motion artifact, or incomplete annular visualization may have introduced selection bias. Fourth, the six geometric strategies were evaluated as parallel exploratory comparisons without formal multiplicity-adjusted superiority testing. Accordingly, although the A4C + A2C elliptical strategy showed the most favorable agreement profile, its apparent advantage should not be interpreted as confirmatory evidence of statistical superiority. Fifth, formal MR severity reclassification was not assessed, so the frequency and clinical consequences of category changes near guideline-defined thresholds remain uncertain. Sixth, all measurements were performed by a single observer, and intraobserver and interobserver reproducibility were not formally evaluated. Finally, only the standard PLAX, A4C, and A2C views were examined, whereas alternative imaging planes or dedicated biplane acquisitions may yield different results. Future prospective, multicenter studies should include broader populations, standardized phase-matched protocols, independent observers, formal reproducibility and reclassification analyses, and external validation of the A4C + A2C elliptical strategy within guideline-based multiparametric MR assessment.

## 5 Conclusion

In summary, this study indicates that Doppler volumetric quantification of MR in older patients is highly sensitive to how the mitral annulus is modeled and which 2D view(s) are used to measure annular diameters. Compared with circular single-diameter strategies, an A4C + A2C elliptical approach improved agreement with both the 3D TEE annular CSA reference and 3D planimetry-based regurgitant indices, with consistent performance across MR mechanisms and severity strata. These findings support method standardization focused on annular geometry as a pragmatic step to improve Doppler-based MR quantification in older adults in contemporary echocardiographic practice.

## Data Availability

The raw data supporting the conclusions of this article will be made available by the authors, without undue reservation.
