# Supplementary material for: Comparison of circular and elliptical geometric models with optimal two-dimensional imaging view for Doppler quantification of mitral regurgitant volume in older patients
Source: Front Cardiovasc Med. 2026 Aug 31;13:1843639. doi: 10.3389/fcvm.2026.1843639 (PMC13572124; doi:10.3389/fcvm.2026.1843639)
Supplement: Supplementary file 1 [file Datasheet1.docx]

Supplementary Table S1. Primary outcome agreement in functional MR (FMR): Doppler vs 3D reference

| 2D strategy (view × model) | RVol bias, mL (mean ± SD) | RVol 95% LoA, mL (lower to upper) | t value | P value | EROA bias, cm² (mean ± SD) | EROA 95% LoA, cm² (lower to upper) | t value | P value |
| --- | --- | --- | --- | --- | --- | --- | --- | --- |
| Circular model |  |  |  |  |  |  |  |  |
| PLAX | −12.2 ± 14.5 | −40.6 to 16.2 | −9.70 | <0.001 | −0.090 ± 0.095 | −0.276 to 0.096 | −10.89 | <0.001 |
| A4C | −2.0 ± 12.2 | −25.9 to 21.9 | −1.89 | 0.061 | −0.012 ± 0.078 | −0.165 to 0.141 | −1.77 | 0.079 |
| A2C | −6.5 ± 13.0 | −32.0 to 19.0 | −5.77 | <0.001 | −0.045 ± 0.082 | −0.206 to 0.116 | −6.32 | <0.001 |
| Elliptical model |  |  |  |  |  |  |  |  |
| PLAX + A4C | −3.2 ± 10.9 | −24.6 to 18.2 | −3.39 | 0.001 | −0.020 ± 0.072 | −0.161 to 0.121 | −3.20 | 0.002 |
| PLAX + A2C | −4.7 ± 11.3 | −26.9 to 17.5 | −4.81 | <0.001 | −0.031 ± 0.074 | −0.176 to 0.114 | −4.83 | <0.001 |
| A4C + A2C | −0.9 ± 10.2 | −20.9 to 19.1 | −1.02 | 0.310 | −0.006 ± 0.067 | −0.137 to 0.125 | −1.03 | 0.305 |

3D, three-dimensional; A2C, apical two-chamber; A4C, apical four-chamber; EROA, effective regurgitant orifice area; FMR, functional mitral regurgitation; LoA, limits of agreement; MR, mitral regurgitation; PLAX, parasternal long-axis; RVol, regurgitant volume; SD, standard deviation.

Supplementary Table S2. Primary outcome agreement in primary/organic MR (PMR): Doppler vs 3D reference (Bland–Altman)

| 2D strategy (view × model) | RVol bias, mL (mean ± SD) | RVol 95% LoA, mL (lower to upper) | t value | P value | EROA bias, cm² (mean ± SD) | EROA 95% LoA, cm² (lower to upper) | t value | P value |
| --- | --- | --- | --- | --- | --- | --- | --- | --- |
| Circular model |  |  |  |  |  |  |  |  |
| PLAX | −9.5 ± 13.5 | −36.0 to 17.0 | −8.16 | <0.001 | −0.070 ± 0.085 | −0.237 to 0.097 | −9.58 | <0.001 |
| A4C | −0.5 ± 11.3 | −22.6 to 21.6 | −0.52 | 0.604 | −0.008 ± 0.072 | −0.149 to 0.133 | −1.29 | 0.200 |
| A2C | −4.8 ± 12.2 | −28.7 to 19.1 | −4.58 | <0.001 | −0.036 ± 0.078 | −0.189 to 0.117 | −5.37 | <0.001 |
| Elliptical model |  |  |  |  |  |  |  |  |
| PLAX + A4C | −2.3 ± 10.1 | −22.1 to 17.5 | −2.65 | 0.009 | −0.016 ± 0.068 | −0.149 to 0.117 | −2.73 | 0.007 |
| PLAX + A2C | −3.5 ± 10.4 | −23.9 to 16.9 | −3.91 | <0.001 | −0.026 ± 0.070 | −0.163 to 0.111 | −4.32 | <0.001 |
| A4C + A2C | −0.3 ± 9.4 | −18.7 to 18.1 | −0.37 | 0.713 | −0.004 ± 0.064 | −0.129 to 0.121 | −0.72 | 0.474 |

3D, three-dimensional; A2C, apical two-chamber; A4C, apical four-chamber; EROA, effective regurgitant orifice area; LoA, limits of agreement; MR, mitral regurgitation; PLAX, parasternal long-axis; PMR, primary mitral regurgitation; RVol, regurgitant volume; SD, standard deviation.

Supplementary Table S3. Stratified analysis by MR severity based on 3D reference EROA (mild/moderate/severe): best-performing strategy (A4C + A2C elliptical)

| Severity stratum | n | RVol bias, mL (mean ± SD) | RVol 95% LoA, mL | t value | P value | EROA bias, cm² (mean ± SD) | EROA 95% LoA, cm² | t value | P value |
| --- | --- | --- | --- | --- | --- | --- | --- | --- | --- |
| Mild | 74 | −0.3 ± 7.0 | −14.0 to 13.4 | −0.37 | 0.714 | −0.002 ± 0.030 | −0.061 to 0.057 | −0.57 | 0.571 |
| Moderate | 132 | −0.6 ± 9.2 | −18.6 to 17.4 | −0.75 | 0.454 | −0.004 ± 0.060 | −0.122 to 0.114 | −0.77 | 0.440 |
| Severe | 62 | −1.4 ± 12.8 | −26.5 to 23.7 | −0.86 | 0.392 | −0.010 ± 0.090 | −0.186 to 0.166 | −0.87 | 0.389 |

3D, three-dimensional; A2C, apical two-chamber; A4C, apical four-chamber; EROA, effective regurgitant orifice area; EROA_3D, three-dimensional color Doppler planimetric effective regurgitant orifice area; LoA, limits of agreement; MR, mitral regurgitation; RVol, regurgitant volume; SD, standard deviation.
